# Acute Hepatitis E Virus infection with coincident reactivation of Epstein-Barr virus infection in an immunosuppressed patient with rheumatoid arthritis: a case report

**DOI:** 10.1186/s12879-015-1146-y

**Published:** 2015-10-29

**Authors:** Detlev Schultze, Bernhard Mani, Günter Dollenmaier, Roland Sahli, Andrea Zbinden, Pierre Alexandre Krayenbühl

**Affiliations:** Center of Laboratory Medicine, Frohbergstrasse 3, 9001 St. Gallen, Switzerland; Institute of Microbiology, Centre Hospitalier Universitaire Vaudois and University of Lausanne, Rue du Bugnon 48, 1011 Lausanne, Switzerland; Institute of Medical Virology, Winterthurerstrasse 190, 8057 Zürich, Switzerland; Spital Linth, Gasterstrasse 25, 8730 Uznach, Switzerland

**Keywords:** Hepatitis E virus, Epstein-Barr virus, Immunosuppression, Rheumatoid arthritis, Methotrexate

## Abstract

**Background:**

Hepatitis E virus (HEV) is the most recently discovered of the hepatotropic viruses, and is considered an emerging pathogen in developed countries with the possibility of fulminant hepatitis in immunocompromised patients. Especially in the latter elevated transaminases should be taken as a clue to consider HEV infection, as it can be treated by discontinuation of immunosuppression and/or ribavirin therapy. To our best knowledge, this is a unique case of autochthonous HEV infection with coincident reactivation of Epstein-Barr virus (EBV) infection in an immunosuppressed patient with rheumatoid arthritis (RA).

**Case presentation:**

A 68-year-old Swiss woman with RA developed hepatitis initially diagnosed as methotrexate-induced liver injury, but later diagnosed as autochthonous HEV infection accompanied by reactivation of her latent EBV infection. She showed confounding serological results pointing to three hepatotropic viruses (HEV, Hepatitis B virus (HBV) and EBV) that could be resolved by detection of HEV and EBV viraemia. The patient recovered by temporary discontinuation of immunosuppressive therapy.

**Conclusions:**

In immunosuppressed patients with RA and signs of liver injury, HEV infection should be considered, as infection can be treated by discontinuation of immunosuppression.

Although anti-HEV-IgM antibody assays can be used as first line virological tools, nucleic acid amplification tests (NAAT) for detection of HEV RNA are recommended – as in our case - if confounding serological results from other hepatotropic viruses are obtained. After discontinuation of immunosuppressive therapy, our patient recovered from both HEV infection and reactivation of latent EBV infection without sequelae.

## Background

Hepatitis E virus (HEV) is a non-enveloped single-stranded RNA virus approximately 27–34 nm in diameter belonging to the genus *Hepevirus* in the *Hepeviridae* family.

It is the most recently discovered of the hepatotropic viruses, with four different HEV genotypes (GT) that represents a single serotype able to infect humans [1]. Though humans are traditionally regarded the only reservoir for GT1 (Asia and North Africa) and GT2, there are reports of GT1 circulating in pigs as well. This zoonotic potential is well-demonstrated for GT3 in the Americas, Europe and Asia, and for GT4 in Asia, where they are also found in pigs and wild animals [2]. It has recently become obvious that HEV is endemic in industrialized countries, and that more infections are autochthonous (locally acquired) than travel-associated [3]. HEV infection has to be considered as a zoonosis and viral transmission from animals (pigs, wild animals) occurs through food or direct contact [1]. In high-income countries of North America and Europe, in Japan and Australia, autochthonous transmission of HEV causes both asymptomatic infections in healthy individuals as well as fulminant hepatitis in mostly immunocompromised patients [4]. Although it is now considered to be an emerging disease, HEV infection is yet not a notifiable disease in Switzerland [5].

We present a unique case of autochthonous HEV infection in a Swiss woman with coincident reactivation of Epstein-Barr virus (EBV) infection under immunosuppression of her RA.

### Case presentation

On April 22nd 2015 a 68-year-old Swiss woman complained about nausea, headache and fever. She had slightly elevated transaminases and elevated C-reactive protein. The general practitioner interpreted the abnormal liver function tests as complication of MTX therapy she had received for treatment of RA since 2002. As transaminases increased over the following eight days to values above upper limit in point-of-care testing, the patient was admitted with preliminary diagnosis of MTX-induced liver injury [6]. On admission the patient complained of nausea, pain below right rib cage and in lower extremities and buttocks. She was afebrile, showed no jaundice and further physical examination was unremarkable. Abdominal ultrasound showed slight liver steatosis. Liver magnetic resonance imaging revealed no further abnormalities. The low-dose MTX therapy (10 mg/week) for erosive RA (positive for anti-cyclic citrullinated peptide-antibody) and the weekly prednisolon dosis (5mg) that she had received for treatment of chronic obstructive lung disease were stopped. The therapy with bronchodilators (ipratropium bromide, budenoside, albuterol) was continued.

Liver transaminases, alkaline phosphatase, gamma-glutamyl transferase, and acute phase proteins (C-reactive protein, ferritin, haptoglobin) were elevated (Table 1). Leukocytes and platelets were slightly above normal reference limit. Bilirubin, prothrombin time, and activated partial thromboplastin time laid within normal ranges. Serology for hepatotropic viruses and parasites (Hepatitis A virus, Hepatitis C virus, Cytomegalo-virus (CMV), *Echinococcus alveolaris*, *Echinococcus granulosus*, *Toxoplasma gondii*) and anti-mitochondrial-antibody as well as markers for autoimmune hepatitis (anti-liver kidney microsome type 1-, anti-smooth muscle-, anti-soluble liver antigen- antibody) were negative.

**Table 1:** Evolution of laboratory parameters and discontinuation of therapy

Parameter (Reference or limit of detection)	Day after onset of illness					
	8	9	12	21	40	75
AST (<40 U/l)	1920	1787	578	53	31	26
ALT(<55 U/l)	1743	1650	1021	122	27	25
yGT(<35 U/l)	265	226	221	102	35	20
ALP (42–98 U/l)	352	296	302	150	70	58
Bilirubin (<20 umol/l)	15	15	13	16		
CRP(<5 mg/l)	41	35	14	37	12	4
CMV IgG (neg)	Neg					
CMV IgM (neg)	Neg					
Hepatitis A IgM (neg)	Neg					
HBs Antigen (neg)	Neg					
HBc Ig (neg)		Pos			Neg	
HBc IgM (neg)		Pos			Neg	
HBe Ig (neg)		Neg			Neg	
HBe Antigen (neg)		Neg				
HBs Ig (neg)		Neg				
HBV DNA (<9 IU/ml)			<9			
Hepatitis C Ig (neg)	Neg					
EBV VCA IgG (neg)		Pos				
EBV NA1 IgG (neg)		Pos				
EBV VCA IgM (neg)		Pos			Pos	
EBV DNA (<122 IU/ml)			566		<122	<122
Heterophile IM (neg)	Neg					
HEV IgG (neg); Diapro		Pos				
HEV IgM (neg); Diapro		Pos				
HEV IgG (<1.0 index); Wantai	5.3				19.4	19.5
HEV IgM (<1.0 index); Wantai	10.4				10.6	10.6
HEV RNA (<1000 cc/ml)				8959	<1000	<1000
Stop (\) and reinitiation (/) of						
- Prednisolon therapy	8 \					/ 48
- Methotrexate therapy	8 \					/ 63

To note, tests for specific IgM-antibody against EBV viral capsid antigen (VCA), Hepatitis B-Virus (HBV)-core-antigen (Architect®, Abbott, Wiesbaden, Germany), and HEV (Diapro, Milan, Italy) were positive, indicating a recent infection with three different viruses. Subsequent viral load testing revealed low copy numbers of EBV DNA (928 copies/ml corresponding to 566 IU/ml calibrated to World Health Organisation EBV standard; TaqMan real-time PCR assay according to Berger et al. [7] with modified primers) and HEV RNA (8959 copies/ml; Real-time reverse transcriptase (RT)--PCR assay according to Jothikumar et al. [8] with minor primer modifications to account for HEV genotypic diversity). No HBV DNA could be detected in plasma (COBAS AmpliPrep-COBAS TaqMan 48 HBV Test, Version 2.0, Roche, Switzerland). Additional HEV IgG- and IgM-specific ELISA tests (Beijing Wantai Biological Pharmacy Enterprise), retrospectively done on a serum sample from day 8 of illness confirmed positive results.

One month follow-up showed clearing of both Hepatitis E- and Epstein-Barr viral load and seroreversion of anti-HB core-IgM and anti-HB core-Ig antibody, with significant increase of HEV IgG index. Results of blood tests are shown in Table 1.

The patient fully recovered after 40 days and suffered no relapse after reinitiation of MTX and prednisolon therapy, in the following five weeks.

## Discussion

Although HEV infection is often asymptomatic and only occasionally induces a self-limited acute hepatitis with low mortality, it can, however, be more severe in immune-suppressed individuals [4]. Acute HEV infection can be diagnosed either directly by detecting the viral genome in biologic specimens (e.g. stools, liver biopsy) or indirectly by detecting specific IgM and IgG antibodies against the virus [2]. In plasma, HEV RNA may be detected at low levels with onset of illness, persisting for up to 4 weeks. In immunosuppressed patients, HEV diagnosis by detection of HEV RNA is recommended, as testing for antibodies may give false negative results due to immunosuppression [2]. The detection of HEV specific-IgG and -IgM can be delayed in immunosuppressed patients e.g. after liver transplantation, with a diagnostic window of several weeks between the first detection of HEV RNA and the detection of HEV-specific antibodies [9]. In blood samples of 35 immunosuppressed solid-organ transplant patients and five haematological patients, drawn at the time of first elevation of the ALT activity and positive for HEV RNA, Abravanel F et al. [10] reported a sensitivity of 85 % (95 % CI: 70.2-94.3) for detection of HEV specific-IgM antibodies (ELISA Kit, Beijing Wantai Biological Pharmacy Enterprise). The authors concluded, that well validated anti-HEV-IgM assays can be used as first line virological tool in immunosuppressed patients, but molecular detection of HEV RNA is essential, if anti-HEV IgM tests turn out negative.

Furthermore, serological testing is hindered either by the potential inability to detect anti-HEV IgM or by false positive HEV-IgM results that have been reported to occur in infections by other hepatotropic viruses like EBV and CMV. In the latter case, false positive HEV-IgM antibodies may appear, e.g., as a consequence of polyclonal B-cell stimulation by EBV infection or – rarely - crossreactive EBV-specific IgM antibodies. Since liver involvement is not rare in both viruses, acute hepatitis may be incorrectly diagnosed as being due to HEV infection or (vice versa) to EBV or CMV infection, respectively, if the diagnosis is not confirmed by molecular tests [11, 12].

Although the classical presentation of acute HEV infection is with general malaise, fever, jaundice accompanied by pale stools, darkened urine and gastrointestinal symptoms, this phenotype is rarely seen with HEV GT3 and 4 in high income countries [2]. Our patient had mild gastrointestinal symptoms, lacking jaundice. In such atypical cases, liver enzyme elevations especially elevated aspartate transaminase (AST) and alanine transaminase (ALT) are the clue for the correct diagnosis, but may be incorrectly ascribed to other acute hepatic diseases such as drug-induced liver injury [2]. In our patient, elevated liver enzymes were initially interpreted as complication of MTX therapy, despite folic acid supplementation that lowers frequency of liver function abnormalities. Although low-dose MTX therapy is considered immunosuppressive, it does not increase infection risk in patients with RA, that per se have a higher rate of infection when compared with a healthy control population [13]. A french retrospective multicenter study [14] demonstrated that HEV infection should be suspected in patients with RA and elevated liver enzyme levels. Among RA patients in this study, HEV infection had been treated by discontinuation of immunosuppressive therapy to allow restoration of T cell responses against the virus and by ribavirin therapy when liver enzyme levels were particularly elevated and/or when the activity of the inflammatory arthritis required immunosuppressive drugs. Nearly half of all patients including other inflammatory rheumatic diseases reported in this study by Bauer et al. were totally asymptomatic, emphasizing the need to specifically look for HEV in the checkup of elevated liver enzymes, regardless of the symptoms [14].

HEV GT3 infection in immunocompromised patients may become chronic, defined as HEV RNA detectable for more than 6 months, leading to chronic hepatic inflammation and rapidly progressive cirrhosis, as in patients chronically infected with hepatitis C virus [2].

In our patient, discontinuation of MTX and prednisolone therapy during one month, i.e., time from hospital admission (day 8) and the day of the first negative PCR result for HEV RNA (day 40), was sufficient to clear both HEV RNA and EBV DNA. This is in the range of the french study [14], in which 9 of 12 patients with RA (7 females; median 61 yr , range 44–72 yr ) received MTX, partially in combination with a biologic (e.g.Rituximab), corticosteroid and/or disease-modifying drug (Leflunomide). After discontinuation of immunosuppressive therapy, the median time of HEV RNA positivity in 10 of these patients was 7 weeks (range 4–10.5 weeks). One 68-year-old man on MTX and Rituximab received ribavirin for 3 months and the HEV load disappeared within 8 weeks. In contrast to our patient, no reactivation of latent EBV infection was noted. In accordance with the study patients, the course of acute HEV in our patient was found to be cytolytic rather than cholestatic, with a duration of liver cytolysis of <40 days, compared to <3 months in study patients. Additionally, our patient experienced no flare of its rheumatic disease after discontinuation of immunosuppression, whereas it occurred in half of the study patients at a median of 4 weeks (range 1 day −10 weeks).

EBV persists as a latent infection with episomal DNA in a very small number of memory B cells, and it is difficult to detect EBV in the plasma of healthy individuals [15, 16], whereas in patients with RA there is a 10-fold systemic EBV overload in peripheral blood mononuclear cells (PBMCs) due to an impaired immune response to EBV [17]. MTX therapy of patients with RA may reactivate EBV production [15] by differentiation of memory B cells into plasma cells that undergo lytic infection and produce virus [16]. In our patient, concomitantly positive EBV VCA-IgG, EBV VCA-IgM, and EBNA-1 IgG antibodies were measured. Although this reflects a past EBV infection in most cases, it may also correspond to an EBV reactivation episode, a state of polyclonal stimulation by a heterologous infectious agent or to a relatively recent EBV-related primary infection [18]. In our patient, the low viral load of EBV and a negative test for heterophile antibodies (Clearview IM; Inverness Medical, UK) pointed against primary EBV infection and rather indicated a reactivation of latent EBV [19]. The negative HBs antigen, anti-HBs-Ig, and anti-HBe-IgG were a hint to falsely positive anti-HB core-IgM and anti-HB core-Ig antibody, confirmed by seroreversion one month later. Taken together, microbiological tests indicated a recent HEV infection with coincident reactivation of the patient’s latent EBV infection.

One month after discontinuation of MTX and prednisolon therapy, neither HEV RNA nor EBV DNA was detectable in the patient’s plasma. Thus, reactivation of the patient’s EBV-infection might have been triggered by the HEV infection.

Most HEV transmissions occur by the fecal-oral (GT1 and GT2) and food-borne routes (GT3 and GT4). HEV has been documented in pig products in the human food chain across Europe, and shellfish, such as bivalve mollusks, have been shown to act as reservoirs [2] Other transmission routes, including human to human transfer [20], vertical transmissions and infection through blood transfusion have also been described [3]. Environmental presence of HEV has been demonstrated in sewage samples of developed countries, where sporadic, locally acquired infections of HEV genotypes 3 and 4 have been reported during the past decade. These autochthonous zoonotic infections have been linked to ingestion of raw or undercooked meat especially from pigs, wild boar, and deer [2]. In several western European countries including Switzerland, seroepidemiological studies suggest that domestic pigs and wild boars are the main reservoirs for human HEV. A significantly lower seroprevalence in domestics pigs in Switzerland was observed in 2011 than in 2006, possibly due to the ban in 2008 on using sewage sludge from water treatment plants as fertilizer in agriculture [21].

As our patient had not left Switzerland for many years, she must have acquired an autochthonous infection, possibly due to consumption of pork spare ribs four weeks prior to onset of symptoms. Genotyping of the HEV was not feasible, due to the low viral load that hampered amplification of cDNA to detectable levels.

## Conclusions

In immunosuppressed patients with RA and signs of liver injury, HEV infection should be considered, as infection can be treated by discontinuation of immunosuppression. Although anti-HEV-IgM antibody assays can be used as first line virological tools, NAAT for detection of HEV RNA are recommended – as in our case - if confounding serological results from other hepatotropic viruses are obtained. After discontinuation of immunosuppressive therapy, our patient recovered from both HEV infection and reactivation of latent EBV infection without sequelae.

## Consent

Written informed consent was obtained from the patient for publication of this case report. A copy of the written consent is available for review by the Editor-in-Chief of this Journal.
